# Correlation of T_2_ NMR with $T_{g}/T$ and viscosity

**DOI:** 10.1016/j.crfs.2026.101309

**Published:** 2026-01-12

**Authors:** Ruud van der Sman, Stefano Renzetti, Panos Voudouris, Ali Asghari, Seddik Khalloufi

**Affiliations:** a Wageningen-Food & Biobased Research, The Netherlands; b Food Process Engineering, Wageningen University & Research, The Netherlands; c Laval University, Quebec, Canada

**Keywords:** T_2_ NMR, Glass transition, Viscosity, Sugar replacement

## Abstract

Using both literature and new experimental data, we demonstrate that $T_{2}$ relaxation times are governed by the ratio $T_{g}/T$, where $T_{g}$ is the moisture-dependent glass transition temperature and T is the actual temperature. This ratio, $T_{g}/T$, is known to control the viscosity and plasticizing behavior of small carbohydrates, as well as the rheological properties of biopolymeric systems such as starch and plant proteins. For small carbohydrates, the $T_{2}$ data collapse onto a universal master curve, analogous to their viscosity behavior. For biopolymers, however, the current dataset is insufficient to confirm the existence of such a master curve. Additionally, we show that $T_{2}$ NMR provides an accurate estimate of the glass transition temperature of small carbohydrates, with deviations within 1%–2%. This makes it a promising method for high-throughput screening of the plasticizing properties of novel sugar replacers.

## Highlights

• Logarithmic T2 relaxation time scales of carbohydrates with T_g_/T.
• Logarithmic T2 relaxation time scales of carbohydrates with viscosity.
• T2 NMR method is an accurate method for determining glass transition.
• T2 NMR is a promising high-throughput method for screening novel sugar replacers.

## Graphical abstract

**Figure:**
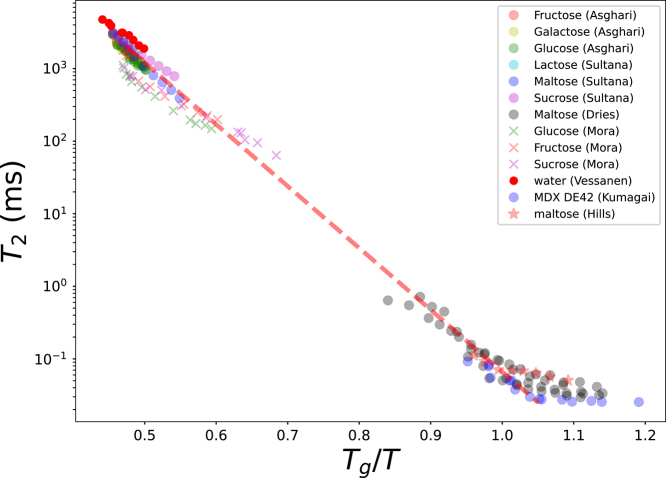

## 1 Introduction

In their study on sugar replacement in cake, Godefroidt et al. (2023) reported a correlation between $T_{2}$ relaxation times (as measured by NMR) and the (intermolecular) hydrogen bond density, $n_{OH,eff}$. The latter parameter is central to our sugar replacement strategy (van der Sman et al., 2022), where we have shown that $n_{OH,eff}$ governs the texture and rheology of bakery products.

Previously, we demonstrated that $n_{OH,eff}$ also controls both the viscosity (Van der Sman and Mauer, 2019) and the glass transition temperature $T_{g}$ (Van Der Sman, 2013) of carbohydrate solutions. Consequently, the ratio $T_{g}/T$—the glass transition temperature over the actual temperature—emerges as a key parameter controlling the viscosity of these systems (Van der Sman and Mauer, 2019, Van der Sman and Meinders, 2013, Longinotti and Corti, 2008). Mora-Gutierrez and Baianu (1989) also proposed that $T_{2}$ relaxation times are linked to intermolecular hydrogen bonding, akin to our $n_{OH,eff}$ parameter. Similarly, Asghari et al. (2025) suggested that $T_{2}$ relaxation times reflect hydrogen bond strength in carbohydrate solutions.

Aschenbrenner et al. (2014) correlated $T_{2}$ relaxation times with the temperature difference $T-T_{g}$, observing that data for lactose solutions collapse onto a single curve, though this was not the case for trehalose or dextran. More broadly, we have found that the temperature ratio $T_{g}/T$ governs the rheological behavior of various food biopolymer systems, including starch, maltodextrins, and plant proteins (Van der Sman et al., 2022, Siemons et al., 2022, van der Sman et al., 2023, van der Sman et al., 2024, Purcell et al., 2025).

Traditionally, the glass transition temperature of food systems is measured using differential scanning calorimetry (DSC) (Renzetti et al., 2021) or dynamic mechanical analysis (DMA) (Veser et al., 2024). However, in complex systems involving biopolymers and co-plasticizers such as sugars, DSC often fails to provide a clear glass transition signal (Masavang et al., 2019). DMA also has limitations in determining $T_{g}$ due to the broad nature of the transition in such systems (Cuq et al., 1997, Hundal and Takhar, 2009, Mendieta-Taboada et al., 2008).

This raises two key questions: (a) can $T_{2}$ relaxation times obtained from low-field NMR serve as an alternative method for determining the $T_{g}/T$ parameter? and (b) do $T_{2}$ relaxation times correlate with the viscosity of food materials where hydrogen bonding dominates molecular interactions?^1^

Independent studies support this link between $T_{2}$ relaxation and rheology. For example, correlations have been observed in protein-rich 3D-printing inks (Chen et al., 2021, Phuhongsung et al., 2020), carbohydrate solutions (Sato and Miyawaki, 2007), and microcrystalline cellulose suspensions (Ono et al., 1998). Sacchetti et al. (2014) also reported a correlation between $T_{2}$ and viscosity in carbohydrate solutions. Ruan et al. (1999) used both $T_{1}$ and $T_{2}$ NMR to determine the glass transition of maltodextrins. In the glassy state $T_{2}$ take a constant value, while $T_{1}$ depends on temperature and moisture content, while in the rubbery state they show the reverse behavior, i.e. a constant $T_{1}$ value and a varying $T_{2}$ value. The transitions in $T_{1}$ an $T_{2}$ are used to determine $T_{g}$. Deng et al. (2014) noted that both $T_{1}$ and $T_{2}$ correlate with viscosity, and that 2D NMR measurements combining $T_{1}$ and $T_{2}$ may offer additional insights.

This relationship between NMR relaxation times and viscosity is a foundational aspect of the theory developed by Bloembergen et al. (1948). Nuclear spin relaxation arises from time-dependent local magnetic field fluctuations, which are generated by molecular rotational and translational motion—modulating dipole–dipole interactions between nuclear spins. In the theory the relaxation times relate to rotational diffusion. For (nearly) spherical molecules this can be linked to viscosity, via the Stokes–Einstein theory.

Given our theoretical framework, in which the viscosity of carbohydrate solutions scales with the temperature ratio $T_{g}/T$, we hypothesize that $T_{2}$ relaxation times should similarly scale with $T_{g}/T$.

As the glass transition behavior of carbohydrates such as sugars is well established (Van der Sman and Mauer, 2019), we have focused our investigation on these systems to test our hypothesis. Specifically, we utilize (1) literature data on $T_{2}$ relaxation times of carbohydrates and pure water, and (2) new experimental data obtained on carbohydrate solutions and biopolymeric samples (soy and maize flour). We examine the correlation between the temperature ratio $T_{g}/T$ and both the $T_{2}$ relaxation time and the viscosity of the carbohydrate solutions.

## 2 Materials and methods

### 2.1 NMR relaxation measurements

The literature data were obtained using benchtop low-field NMR analyzers, employing either Free Induction Decay (FID) or Carr–Purcell–Meiboom–Gill (CPMG) pulse sequences (van den Dries et al., 1998, Mora-Gutierrez and Baianu, 1989, Asghari et al., 2024, Sultana et al., 2024). Continuous $T_{2}$ relaxation time spectra were derived using the standard inverse Laplace transform method (CONTIN). In our analysis, we considered only the $T_{2}$ relaxation time corresponding to the main peak (i.e., the signal with the largest amplitude).

For our own NMR measurements, we used a 20 MHz minispec mq 20 benchtop NMR spectrometer (Bruker, Germany). Carbohydrate samples were analyzed using a CPMG pulse sequence with an echo time of 0.2 ms and a train of 32,000 echoes. The number of scans was set to 4, with a recycle delay of 30 s. Background correction was performed using measurements from an empty sample tube. Each CPMG dataset was reduced to 2000 geometrically spaced data points before fitting the decay curves using CONTIN.

For biopolymer samples, a combined FID-CPMG sequence was used. The FID acquisition time was 0.1 ms, followed by a CPMG pulse train with an echo time of 0.2 ms and 1000 data points. The number of scans was set to 8, with a recycle delay of 8 s. Each sample was measured in triplicate. Measurements were conducted after incubation at 20, 40, and 60 °C for 30 min, respectively.

Differences between datasets may arise due to variations in magnetic field strength (B), or equivalently, angular frequency (ω), which are linearly related via the Larmor resonance frequency:

$$\omega =\gamma_{H}B,$$

where $\gamma_{H}=2.675\times 10^{8}$ rad/s/T for ${}^{1}$H protons. A 20 MHz spectrometer typically corresponds to a magnetic field strength of approximately 0.5 T.

The Bloembergen–Purcell–Pound (BPP) relation (Bloembergen et al., 1948) describes the dependence of $T_{2}$ on molecular motion:

$$\frac{1}{T_{2}}=C\left[a\tau_{c}+b\frac{\tau_{c}}{1+{\left(\omega \tau_{c}\right)}^{2}}\right],$$

where $\tau_{c}$ is the correlation time of molecular motion. At low magnetic fields, where $\omega \tau_{c}\ll 1$, the relation simplifies to $1/T_{2}∼\tau_{c}$. Since $\tau_{c}$ is expected to follow the same temperature and moisture dependence as viscosity (Zielinski et al., 1992), a logarithmic relationship between $T_{2}$ and $T_{g}/T$ is anticipated, i.e., $\log\left(T_{2}\right)∼T_{g}/T$, similar to the relations between $T_{g}/T$ and rheological properties of carbohydrate solutions, polysaccharides and proteins (Van der Sman and Mauer, 2019, van der Sman et al., 2023, Van der Sman et al., 2022).

### 2.2 Computation of glass transition

The glass transition temperature $T_{g}$ of carbohydrate solutions is calculated using the Couchman–Karasz equation:

(1) $$T_{g}=\frac{y_{w}T_{g,w}\Delta C_{p,w}+y_{s}T_{g,s}\Delta C_{p,s}}{y_{w}\Delta C_{p,w}+y_{s}\Delta C_{p,s}}$$

where $y_{w}=1-y_{s}$ is the mass fraction of water, $T_{g,w}=139$ K is the glass transition temperature of pure water, and $T_{g,s}$ is the glass transition temperature of the dry solute. Mass fractions are readily calculated from the set concentrations of the investigated carbohydrate solutions.

The specific heat capacity changes during the glass transition are $\Delta C_{p,w}=1.92$ kJ/kg for water, $\Delta C_{p,s}=0.425$ kJ/kg for sugars, and $\Delta C_{p,s}=0.85$ kJ/kg for polyols and amino-acids (Van Der Sman, 2013, Van der Sman et al., 2020). The latter value has been found to be universal for carbohydrates with molar masses $M_{w}\geq 180$ g/mol (Van Der Sman, 2013). Values for $T_{g,s}$ of various carbohydrates are tabulated in Van der Sman and Mauer (2019).

**Table 1:** Data sources used for the correlation of T2 with $T_{g}/T$.

Compound	Reference
Glucose, fructose, sucrose	Mora-Gutierrez and Baianu (1989)
water	Vesanen et al. (2013)
Fructose, Glucose, Galactose	Asghari et al. (2024)
Lactose, Maltose, Sucrose	Sultana et al. (2024)

Maltose	van den Dries et al. (1998)
maltodextrin DE42	Kumagai and Kumagai (2002)
Maltose	Hills and Pardoe (1995)

## 3 Results

### 3.1 $T_{2}$ Relaxation times of carbohydrates

We first collected $T_{2}$ relaxation time data for carbohydrate systems and pure water from the literature, as summarized in Table 1. Several sources (Hills and Pardoe, 1995, van den Dries et al., 1998, Kumagai and Kumagai, 2002) are of particular interest, as they report data for systems near or within the glassy state, i.e. $T_{g}/T>1$. The remaining datasets pertain to carbohydrate solutions in the rubbery or liquid-like state, i.e. $T_{g}/T<1$.

All collected data were correlated with the estimated $T_{g}/T$ ratio, where T is the temperature at which the NMR measurement was performed, and $T_{g}$ is the moisture-dependent glass transition temperature of the carbohydrate solution. The glass transition temperature $T_{g}$ was computed using the Couchman–Karasz relation (Eq. (1)), employing tabulated values of the dry solute glass transition temperature $T_{g,s}$ from our previous work (Van der Sman and Mauer, 2019). These $T_{g,s}$ values are primarily based on DSC measurements.

**Fig. 1:**
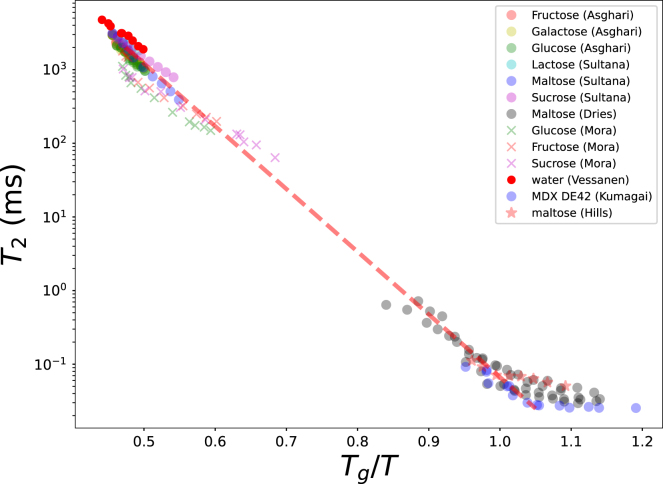
$T_{2}$ relaxation time of various carbohydrate solutions and glasses, with data collected from literature as listed in Table 1. The dashed line indicates the fitted trend line connecting the values of pure water at room temperature, and the universal mobility state of carbohydrate systems in the glassy state (with $T_{g}/T=1.05$).

Fig. 1 presents the literature $T_{2}$ data as a function of the estimated $T_{g}/T$. The results are highly promising: $\log\left(T_{2}\right)$ exhibits a linear relationship with $T_{g}/T$ in the range $T_{g}/T<1$. For $T_{g}/T>1.05$, the relaxation time plateaus at a universal value of approximately 0.02 ms. This limiting value is consistent with findings by Kalichevsky et al. (1993) and Aschenbrenner et al. (2014).

The observed plateau can be interpreted through the isoviscosity assumption of the glassy state (Angell, 2002), which posits a universal viscosity $\eta =\eta_{g}\approx 10^{13}$ Pa⋅s. Notably, the leveling off of $T_{2}$ values does not occur precisely at $T_{g}/T=1$, but rather at $T_{g}/T=1.05$. This aligns with previous observations that the $T_{g}$ determined via $T_{2}$ NMR is approximately 30 °C higher than that obtained from DSC (Kalichevsky et al., 1992, Kalichevsky et al., 1993, Yoshioka et al., 1999).

Based on the universality of the $T_{2}$ value in the glassy state and the well-established $T_{2}$ value of pure water, we have included a trend line in Fig. 1. This trend-line was obtained via linear regression between $T_{g}/T$ and $\log\left(T_{2}\right)$ in the range $T_{g}/T<1.0$. This rendered a strong correlation of $r^{2}=0.996$. Extrapolation of the trend line to the plateau value of $T_{2}$=0.02 ms, confirms the limiting value of $T_{g}/T=1.05$. Despite this high correlation, we note a significant gap in the collected dataset in the range $0.7<T_{g}/T<0.9$. The occurrence of this gap is probably explained by the solubility limitations of plasticizers in aqueous solutions, which are limited to the range $0.4<T_{g}/T<0.7$. In the range $T_{g}/T>0.9$ shelf-stable powders at low water activities are used. Still, we encourage researchers to produce data to fill this gap.

This trend line can be used for comparison with new experimental data, or to establish their glass transition temperature $T_{g,s}$. As an exercise, we estimate those for glucose and fructose in the next section.

### 3.2 Glass transition of glucose and fructose

In our earlier study (Van der Sman and Mauer, 2019), we assumed that the dry glass transition temperatures ($T_{g,s}$) of fructose and glucose are similar. However, several studies suggest that this may not be the case (Arvanitoyannis et al., 1993, Cruz et al., 2001, Saavedra-Leos et al., 2012, Schugmann and Foerst, 2022). Table 4 lists various reported values of $T_{g,s}$ for fructose and glucose from the literature. To evaluate the accuracy of $T_{2}$-NMR in determining $T_{g,s}$, we analyzed the $T_{2}$ relaxation data from Asghari et al. (2024) for both sugars. We note, that these measurements are done at constant temperature (T=32 ° C).

If fructose and glucose indeed have similar $T_{g,s}$ values, their viscosity data should overlap. To test this, we compiled viscosity data from the literature (Table 2). We observe that for $T_{g}/T<0.6$, the viscosity data of fructose and glucose are nearly identical. However, near the glassy state, their viscosities begin to diverge (Ollett and Parker, 1990). If $T_{g,s}$ values are accurately determined, plotting viscosity against $T_{g}/T$ should yield overlapping curves.

To construct a reliable master curve for viscosity, we also included data for sucrose and trehalose, for which such a master curve has already been demonstrated by Longinotti and Corti (2008). Sucrose data from NBS (Swindells et al., 1958) serve as a reference due to their high accuracy. Following Van der Sman and Mauer (2019), we used $T_{g,s}=336$ K for sucrose and $T_{g,s}=388$ K for trehalose.

**Fig. 2:**
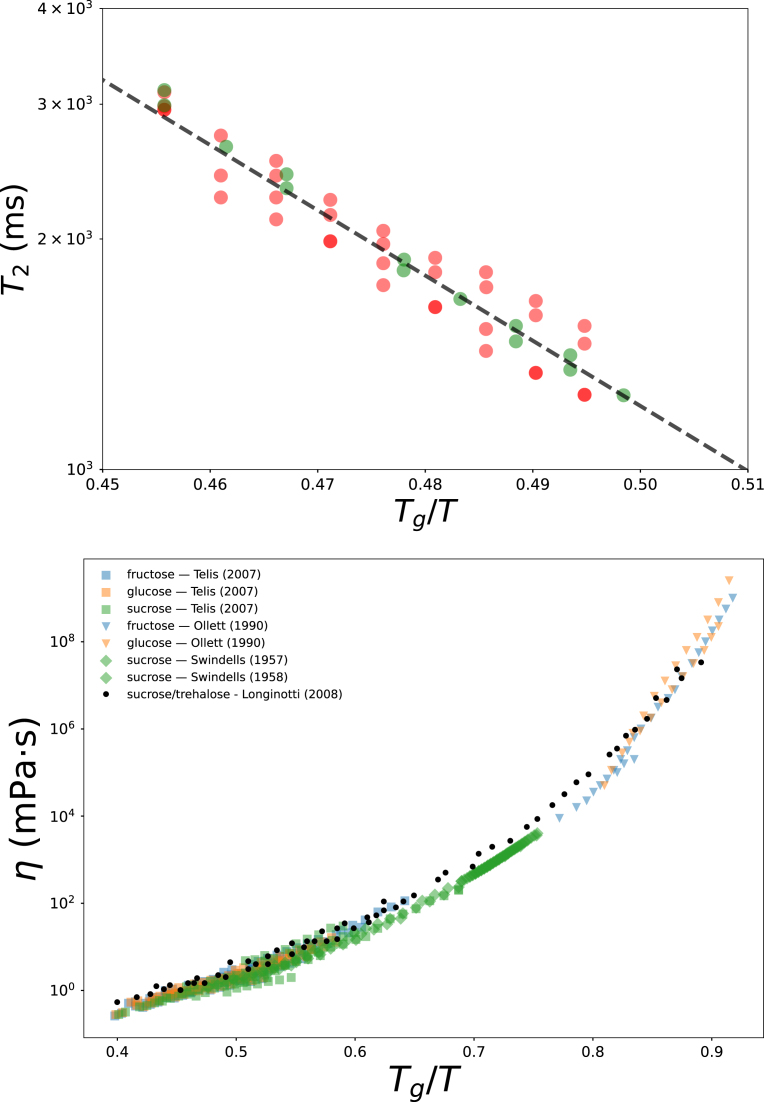
Top: Correlation of $T_{2}$ relaxation time with $T_{g}/T$ for glucose (red) and fructose (green) after their correction of $T_{g,s}$. Bottom:Master curve of viscosity from glucose, fructose, sucrose and trehalose as function of $T_{g}/T$, using the corrected $T_{g,s}$.

By fitting the $T_{2}$-NMR data from Asghari et al. (2024) to the established trendline using least-squares regression, we estimated $T_{g,s}=300.1\pm 6.1$ K for glucose and $T_{g,s}=286.4\pm 3.7$ K for fructose. These estimates yield $T_{2}$ values that closely follow the trendline, as shown in the top panel of Fig. 2. The uncertainties were derived from the covariance matrix of the fit. Using these updated $T_{g,s}$ values, we replotted the viscosity data of glucose and fructose against $T_{g}/T$ (bottom panel of Fig. 2), which now align well with the master curve defined by the disaccharide data. These results increase the confidence in the accuracy of the trend-line.

**Table 2:** Viscosity data sources.

Compound	Reference
Fructose	Ollett and Parker (1990)
Fructose	Telis et al. (2007)
Glucose	Ollett and Parker (1990)
Glucose	Telis et al. (2007)
Sucrose	Telis et al. (2007)
Sucrose	Swindells et al. (1958)
Sucrose	Longinotti and Corti (2008)
Trehalose	Longinotti and Corti (2008)

**Table 3:** Carbohydrates used in new $T_{2}$ measurements.

Compound	$T_{g,s}$ (K)	Reference	RSME
Erythritol	225	Van der Sman and Mauer (2019)	0.046
Mannitol	266	Van der Sman and Mauer (2019)	0.065
Arabinose	274	Van der Sman and Mauer (2019)	0.077
Allulose	274	Allan and Mauer (2022)	0.067
Fructose	286	this paper	0.048
Sucrose	336	Van der Sman and Mauer (2019)	0.087
Lactose	354	Van der Sman and Mauer (2019)	0.105
Trehalose	388	Van der Sman and Mauer (2019)	0.118
Promitor	425	Woodbury and Mauer (2022)	0.262
Nutriose	422	Renzetti et al. (2025)	0.094
Cellobiose	375	Thorat et al. (2018)	0.098
Proline	250	Van der Sman et al. (2020)	0.021
FOS IQ	372	Renzetti et al. (2025)	0.116
Polydextrose (PDX)	353	Renzetti et al. (2025)	0.097
XOS	375	Renzetti et al. (2026)	0.138
FOS Actilight	315	Renzetti et al. (2025)	0.089
FOS OFP	328	Renzetti et al. (2025)	0.071

### 3.3 Analysis of new experimental data

**Fig. 3:**
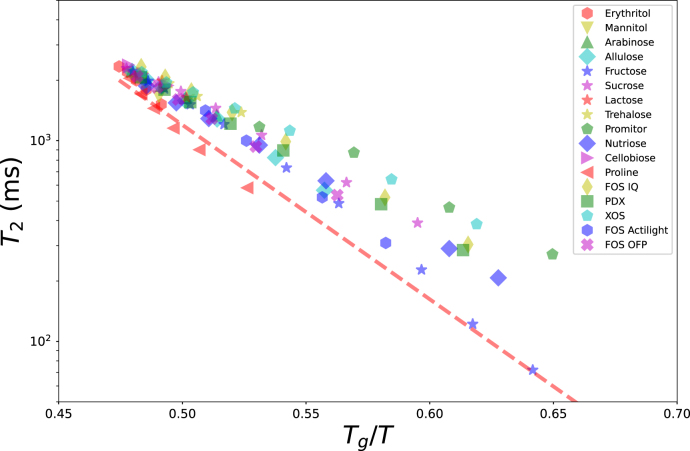
Newly obtained experimental data of T2 relaxation times of carbohydrate systems versus $T_{g}/T$, as compared to the trend line (red dashed line), as fitted to data of Fig. 1.

Subsequently, we analyzed the new experimental data on carbohydrate solutions. The tested carbohydrate solutions are listed in table 3, together with their $T_{g,s}$ values used, as reported in the indicated references. The $T_{g}$ values are computed using the listed $T_{g,s}$ values, mass fractions and Eq. (1). Their $T_{2}$ values versus $T_{g}/T$ are shown in Fig. 3, together with the trend-line from Fig. 1.

We compare the new experimental data with the trend-line using a Root-Mean-Square-Error metric:

(2) $$RMSE=\frac{\underset{i}{\sum }{\left(\overset{ˆ}{y}-y_{i}\right)}^{2}}{N}$$

With $y_{i}=\log\left(T_{2}\right)$, and $\overset{ˆ}{y}$ its prediction based on the trendline. N is the number of datapoints per compound. The RMSE per compound is listed in Table 3.

The RMSE shows that most of the data follow closely the trend line, but there are some strong deviations, especially for novel sugar replacers like Promitor and XOS, whose $T_{g,s}$ is not firmly established yet. Furthermore, in the new experimental data, there appears to be a systematic deviation from the trend-line, which possibly can be attributed to differences in field-strength of the NMR equipment, measurement protocol, or data-analysis techniques used by different sources. We think it is currently best practice to establish trendlines per NMR machine, using samples across the $T_{g}/T$ regime, especially where $0.7<T_{g}/T<0.9$.

### 3.4 $T_{2}$ Relaxation times of biopolymers

**Fig. 4:**
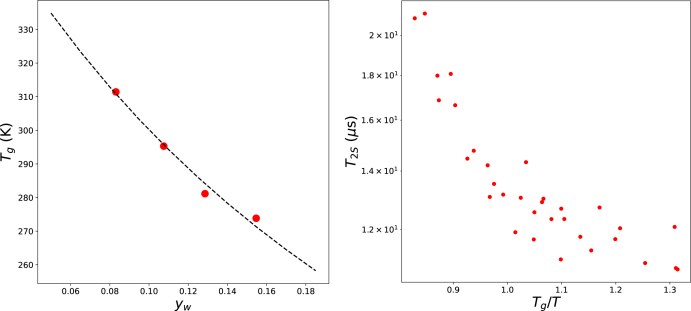
Left pane shows the glass transition of maltodextrin, as measured by Ruan. The solid line indicated the fitting of the data with the Couchman–Karasz theory. The right pane shows the $T_{2S}$-relaxation time as function of $T_{g}/T$, with $T_{g}$ computed using Couchman–Karasz with the given moisture content $y_{w}$.

$T_{2}$-NMR measurements of relatively large maltodextrins (with a dextrose equivalence DE=15) have been reported by Ruan et al. (1998), who also measured the glass transition temperature of this compound using DSC. The corresponding glass transition and NMR data, plotted against $T_{g}/T$, are shown in Fig. 4. Fitting the glass transition data using the Couchman–Karasz equation yielded a dry solute glass transition temperature of $T_{g,s}=382$ K, which was used to compute the $T_{g}/T$ values.

Due to the low moisture content ($y_{w}<0.2$), the dominant NMR signal corresponds to the short spin–spin relaxation time, $T_{2S}$, associated with the mobility of carbohydrate protons (Li et al., 1996). These relaxation times are on the order of microseconds—significantly shorter than the $T_{2}$ values discussed in the previous section. This discrepancy may also stem from the data processing method used by Ruan et al. (1998), who fitted a sum of Gaussians to the raw signal rather than the more conventional sum of exponential decays applied to FID data.

Subsequently, we analyzed our own $T_{2}$ relaxation time data for maize flour and soy flour at varying moisture contents and temperatures. Assuming that the dry glass transition temperatures ($T_{g,s}$) of maize and soy flours are equivalent to those of pure starch (Van der Sman and Meinders, 2011) and pure soy protein van der Sman et al. (2023), we computed $T_{g}$ using the Couchman–Karasz equation. The raw NMR signal was fitted using a bi-exponential decay model, yielding a short ($T_{21}$) and a long ($T_{22}$) relaxation time. The component with the shorter relaxation time, $T_{21}$, consistently exhibited the largest amplitude.

Our analysis shows that $T_{21}$ and $T_{22}$ are of similar magnitude for both maize and soy flour at comparable $T_{g}/T$ values. Because of its lower amplitude, the $T_{22}$ relaxation times are understandably more inaccurate (Callaghan, 1993). Still, the data for maize flour clearly exhibit a plateau in $T_{22}$ at $T_{g}/T>1$, consistent with the behavior observed in the literature.

**Fig. 5:**
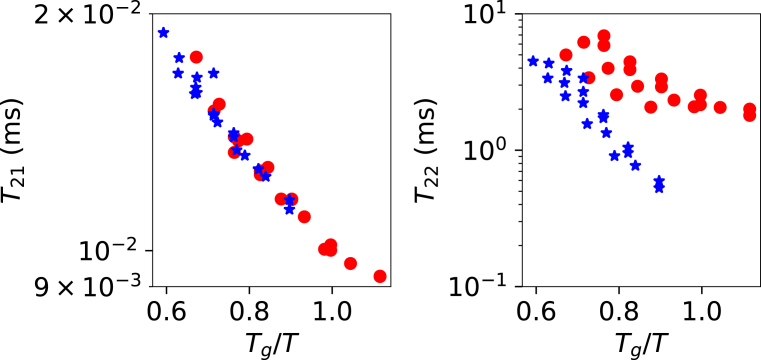
Slow ($T_{21}$) and fast ($T_{22}$) NMR relaxation times of maize (red) and soy bean flours (blue) as function of $T_{g}/T$.

These flours contain raw starches and cellulosic materials, part of which is assumed to be crystalline and thus non-hygroscopic, contributing neither to water absorption nor to the glass transition. We calibrated the degree of crystallinity such that the $T_{22}$ plateau for maize flour aligns with $T_{g}/T=1.05$. For soy flour, the degree of crystallinity was adjusted to align the $T_{21}$ vs. $T_{g}/T$ curves of both flours. The resulting calibrated degrees of crystallinity were ζ=0.15 for soy flour and ζ=0.20 for maize flour. Fig. 5 presents the results after this calibration.

From Fig. 5, we observe that the $T_{21}$ values are comparable to the $T_{2S}$ values of maltodextrin DE15, as discussed earlier. The slope of $\log\left(T_{21}\right)$ versus $T_{g}/T$ is nearly identical for maize and soy flour. Interestingly, $T_{22}$ also appears to correlate with $T_{g}/T$, despite the higher noise level. In particular, the soy flour data suggest a linear relationship between $\log\left(T_{21}\right)$ and $T_{g}/T$ in the range $T_{g}/T<1$. Although the maize flour data are noisier, they also indicate a plateau in $T_{22}$ at $T_{g}/T>1$.

We must note that the investigated biopolymer systems are multicomponent and semicrystalline, that was not fully characterized. The multicomponent character can lead to multiple peaks with different relaxation times, similar to the occurrence of multiple glass transitions in complex mixtures (Duval et al., 2016, Van der Sman, 2019). Peaks associated with different biopolymers as proteins, starch and cellulose may even overlap, given the similarity of relaxation times of soy and maize flours. Crystalline domains can still interact with water via hydrogen bonds (Kulasinski et al., 2015), which can lead to a new water population with their own relaxation time.

**Table 4:** Glass transition temperature $T_{g,s}$ of fructose and glucose following literature.

Compound	$T_{g,s}$ (K)	Reference
Fructose	278	Constantin et al. (2016)
	284	Arvanitoyannis et al. (1993)
	287	Saavedra-Leos et al. (2012)
	280	Orford et al. (1990)
	286	Finegold et al. (1989)
	289	Simatos et al. (1996)

Glucose	304	Constantin et al. (2016)
	310	Saavedra-Leos et al. (2012)
	311	Orford et al. (1990)
	312	Finegold et al. (1989)Schugmann and Foerst (2022)
	314	Arvanitoyannis et al. (1993)

## 4 Conclusion

This study confirms the findings of Godefroidt et al. (2023), demonstrating that $T_{2}$ relaxation time is a convenient and effective method for characterizing the plasticizing properties of sugar replacers. The relaxation time can be directly related to the hydrogen bond density $n_{OH,eff}$ or its associated parameter $T_{g}/T$, the ratio of the glass transition temperature to the actual temperature. For the range $T_{g}/T<1$, we observed a universal linear relationship between $\log\left(T_{2}\right)$ and $T_{g}/T$, which holds across a wide range of plasticizers, including carbohydrate, pure water and proline solutions. For $T_{g}/T>1.05$, the $T_{2}$ relaxation time reaches a constant, universal value.

For glucose and fructose, we tested the accuracy of $T_{2}$ NMR in determining the dry glass transition temperature $T_{g,s}$. By mapping $T_{2}$ values of sugar solutions at various concentrations onto the established $T_{2}$ vs. $T_{g}/T$ trend line, we were able to estimate $T_{g,s}$ with an accuracy of 1%–2%. This demonstrates that $T_{2}$ NMR can be used as a rapid screening tool to estimate the plasticizing properties, glass transition temperature, and viscosity of novel sugar replacers. Moreover, $T_{2}$ NMR is well-suited for integration into high-throughput analytical platforms.

Similar trends were observed for biopolymeric systems, although the $T_{2}$ versus $T_{g}/T$ curves do not overlap with those of small carbohydrate systems. Our findings suggests that while the underlying mechanism may be similar, the specific relaxation behavior may differs due to the more complex molecular architecture, and the semicrystallinity of biopolymers, and warrant further investigations.

Our findings also highlight the potential of $T_{2}$ NMR for determining $T_{g}/T$ in biopolymeric systems, where this ratio is a key parameter governing rheological behavior. However, the current dataset is insufficient to construct a universal master curve, as was achieved for small carbohydrate systems. A promising direction for future research is the study of complex mixtures of biopolymers, water, and secondary plasticizers such as sugars. These systems are prone to phase separation, and the impact of such phenomena on rheology remains poorly understood. We propose that $T_{2}$ NMR could serve as a valuable tool for elucidating these effects.

## CRediT authorship contribution statement

**Ruud van der Sman:** Conceptualization – Writing: first draft; editing + review – Data Analysis. **Stefano Renzetti:** Funding acquisition, Writing – editing + review. **Panos Voudouris:** Data Analysis. **Ali Asghari:** Data curation. **Seddik Khalloufi:** Funding acquisition, Writing – editing + review.

## Declaration of competing interest

Authors declare there is no conflict of interest

## Footnotes

1 Note, that the same symbol T is used for temperature and relaxation time, in agreement with common practice in physics and NMR literature. To avoid any ambiguity, the relaxation times will always be denoted with subscripts, like $T_{1}$ and $T_{2}$, and temperature is denoted without subscripts, and often in the combination $T_{g}/T$.

## References

[b1] Allan M.C., Mauer L.J. (2022). Variable effects of twenty sugars and sugar alcohols on the retrogradation of wheat starch gels. Foods.

[b2] Angell C. (2002). Liquid fragility and the glass transition in water and aqueous solutions. Chem. Rev..

[b3] Arvanitoyannis I., Blanshard J., Izzard M., Lillford P., Ablett S. (1993). Calorimetric study of the glass transition occurring in aqueous glucose: fructose solutions. J. Sci. Food Agric..

[b4] Aschenbrenner M., Grammueller E., Kulozik U., Foerst P. (2014). The contribution of the inherent restricted mobility of glassy sugar matrices to the overall stability of freeze-dried bacteria determined by low-resolution solid-state 1 H-NMR. Food Bioprocess Technol..

[b5] Asghari A., Sultana A., Khalloufi S. (2024). Low-field nuclear magnetic resonance relaxometry: a new approach for monosaccharide identification in sugar solutions. Int. J. Food Sci. Technol..

[b6] Asghari A., Sultana A., Khalloufi S. (2025). Basic and practical analysis of LF-NMR and straightforward toolbox to its successful operation. Appl. Food Res..

[b7] Bloembergen N., Purcell E.M., Pound R.V. (1948). Relaxation effects in nuclear magnetic resonance absorption. Phys. Rev..

[b8] Callaghan P.T. (1993).

[b9] Chen H.z., Zhang M., Yang C.h. (2021). Comparative analysis of 3D printability and rheological properties of surimi gels via LF-NMR and dielectric characteristics. J. Food Eng..

[b10] Constantin J.G., Schneider M., Corti H.R. (2016). Glass transition temperature of saccharide aqueous solutions estimated with the free volume/percolation model. J. Phys. Chem. B.

[b11] Cruz I.B., Oliveira J.C., MacInnes W.M. (2001). Dynamic mechanical thermal analysis of aqueous sugar solutions containing fructose, glucose, sucrose, maltose and lactose. Int. J. Food Sci. Technol..

[b12] Cuq B., Gontard N., Guilbert S. (1997). Thermal properties of fish myofibrillar protein-based films as affected by moisture content. Polymer.

[b13] Deng F., Xiao L., Liao G., Zong F., Chen W. (2014). A new approach of two-dimensional the NMR relaxation measurement in flowing fluid. Appl. Magn. Reson..

[b14] van den Dries I.J., van Dusschoten D., Hemminga M.A. (1998). Mobility in maltose- water glasses studied with 1H NMR. J. Phys. Chem. B.

[b15] Duval A., Molina-Boisseau S., Chirat C., Morel M.H. (2016). Dynamic mechanical analysis of the multiple glass transitions of plasticized wheat gluten biopolymer. J. Appl. Polym. Sci..

[b16] Finegold L., Franks F., Haltey R.H. (1989). Glass/rubber transitions and heat capacities of binary sugar blends. J. Chem. Soc. Faraday Trans. 1: Phys. Chem. Condens. Phases.

[b17] Godefroidt T., Riley I.M., Ooms N., Bosmans G.M., Brijs K., Delcour J.A. (2023). Sucrose substitution in cake systems is not a piece of cake. Npj Sci. Food.

[b18] Hills B., Pardoe K. (1995). Proton and deuterium NMR studies of the glass transition in a 10% water-maltose solution. J. Mol. Liq..

[b19] Hundal J., Takhar P.S. (2009). Dynamic viscoelastic properties and glass transition behavior of corn kernels. Int. J. Food Prop..

[b20] Kalichevsky M., Jaroszkiewicz E., Ablett S., Blanshard J., Lillford P. (1992). The glass transition of amylopectin measured by DSC, DMTA and NMR. Carbohydr. Polymers.

[b21] Kalichevsky M., Jaroszkiewicz E., Blanshard J. (1993). A study of the glass transition of amylopectin—sugar mixtures. Polymer.

[b22] Kulasinski K., Guyer R., Derome D., Carmeliet J. (2015). Water adsorption in wood microfibril-hemicellulose system: Role of the crystalline–amorphous interface. Biomacromolecules.

[b23] Kumagai H., Kumagai H. (2002). Analysis of molecular or ion mobility in glassy and rubbery foods by electric and proton-NMR measurements. Food Sci. Technol. Res..

[b24] Li S., Dickinson L., Chinachoti P. (1996). Proton relaxation of starch and gluten by solid-state nuclear magnetic resonance spectroscopy. Cereal Chem..

[b25] Longinotti M.P., Corti H.R. (2008). Viscosity of concentrated sucrose and trehalose aqueous solutions including the supercooled regime. J. Phys. Chem. Ref. Data.

[b26] Masavang S., Roudaut G., Champion D. (2019). Identification of complex glass transition phenomena by DSC in expanded cereal-based food extrudates: Impact of plasticization by water and sucrose. J. Food Eng..

[b27] Mendieta-Taboada O., Sobral P.J.d.A., Carvalho R.A., Habitante A.M.B. (2008). Thermomechanical properties of biodegradable films based on blends of gelatin and poly (vinyl alcohol). Food Hydrocolloids.

[b28] Mora-Gutierrez A., Baianu I.C. (1989). Proton NMR relaxation and viscosity measurements on solutions and suspensions of carbohydrates and starch from corn: the investigation of carbohydrate hydration and stereochemical and aggregation effects in relation to oxygen-17 and carbon-13 NMR data for carbohydrate solutions. J. Agricult. Food Chem..

[b29] Ollett A.L., Parker R. (1990). The viscosity of supercooled fructose and its glass transition temperature. J. Texture Stud..

[b30] Ono H., Yamada H., Matsuda S., Okajima K., Kawamoto T., Iijima H. (1998). 1H-NMR relaxation of water molecules in the aqueous microcrystalline cellulose suspension systems and their viscosity. Cellulose.

[b31] Orford P.D., Parker R., Ring S.G. (1990). Aspects of the glass transition behaviour of mixtures of carbohydrates of low molecular weight. Carbohydr. Res..

[b32] Phuhongsung P., Zhang M., Devahastin S. (2020). Investigation on 3D printing ability of soybean protein isolate gels and correlations with their rheological and textural properties via LF-NMR spectroscopic characteristics. Lwt.

[b33] Purcell T., Delaplace G., Riaublanc A., Demême M., Derensy A., Della Valle G. (2025). A general viscosity model for high moisture extrudates of pea protein isolates/gluten blend. Phys. Fluids.

[b34] Renzetti S., van den Hoek I.A., van der Sman R.G. (2021). Mechanisms controlling wheat starch gelatinization and pasting behaviour in presence of sugars and sugar replacers: Role of hydrogen bonding and plasticizer molar volume. Food Hydrocolloids.

[b35] Renzetti S., Kan L., Henket J., van der Sman R. (2026). High-throughput viscosity and time-domain 1H NMR relaxometry to determine the plasticizing properties of sugars and polysaccharides solutions. Food Hydrocolloids.

[b36] Renzetti S., Lambertini L., Mocking-Bode H.C., van der Sman R.G. (2025). Soluble fibres modulate dough rheology and gluten structure via hydrogen bond density and flory-huggins water interaction parameter. Curr. Res. Food Sci..

[b37] Ruan R., Long Z., Chen P., Huang V., Almaer S., Taub I. (1999). Pulse NMR study of glass transition in maltodextrin. J. Food Sci..

[b38] Ruan R.R., Long Z., Song A., Chen P.L. (1998). Determination of the glass transition temperature of food polymers using low field NMR. LWT-Food Sci. Technol..

[b39] Saavedra-Leos M., Grajales-Lagunes A., González-García R., Toxqui-Terán A., Pérez-García S., Abud-Archila M., Ruiz-Cabrera M. (2012). Glass transition study in model food systems prepared with mixtures of fructose, glucose, and sucrose. J. Food Sci..

[b40] Sacchetti G., Neri L., Laghi L., Capozzi F., Mastrocola D., Pittia P. (2014). Multidisciplinary approach to study the effect of water status and mobility on the activity of peroxidase in solutions. Food Chem..

[b41] Sato Y., Miyawaki O. (2007). Relationship between proton NMR relaxation time and viscosity of saccharide solutions. Food Sci. Technol. Res..

[b42] Schugmann M., Foerst P. (2022). Systematic investigation on the glass transition temperature of binary and ternary sugar mixtures and the applicability of Gordon–Taylor and couchman–Karasz equation. Foods.

[b43] Siemons I., Veser J., Boom R., Schutyser M., van der Sman R. (2022). Rheological behaviour of concentrated maltodextrins describes skin formation and morphology development during droplet drying. Food Hydrocolloids.

[b44] Simatos D., Blond G., Roudaut G., Champion D., Perez J., Faivre A. (1996). Influence of heating and cooling rates on the glass transition temperature and the fragility parameter of sorbitol and fructose as measured by DSC. J. Therm. Anal..

[b45] Van der Sman R. (2019). Phase separation, antiplasticization and moisture sorption in ternary systems containing polysaccharides and polyols. Food Hydrocolloids.

[b46] van der Sman R., Chakraborty P., Hua N., Kollmann N. (2023). Scaling relations in rheology of proteins present in meat analogs. Food Hydrocolloids.

[b47] Van der Sman R., Van den Hoek I., Renzetti S. (2020). Sugar replacement with zwitterionic plasticizers like amino acids. Food Hydrocolloids.

[b48] van der Sman R., Jurgens A., Smith A., Renzetti S. (2022). Universal strategy for sugar replacement in foods?. Food Hydrocolloids.

[b49] Van der Sman R., Mauer L.J. (2019). Starch gelatinization temperature in sugar and polyol solutions explained by hydrogen bond density. Food Hydrocolloids.

[b50] Van der Sman R., Meinders M. (2011). Prediction of the state diagram of starch water mixtures using the Flory–Huggins free volume theory. Soft Matter.

[b51] Van der Sman R., Meinders M. (2013). Moisture diffusivity in food materials. Food Chem..

[b52] Van der Sman R., Ubbink J., Dupas-Langlet M., Kristiawan M., Siemons I. (2022). Scaling relations in rheology of concentrated starches and maltodextrins. Food Hydrocolloids.

[b53] van der Sman R., Voudouris P., Hamoen J. (2024). Extrapolation of classical rheometry of plant protein pastes to extrusion conditions. Food Hydrocolloids.

[b54] Sultana A., Asghari A., Khalloufi S. (2024). A straightforward method for disaccharide characterization from transverse relaxometry using low-field time-domain nuclear magnetic resonance. Food Anal. Methods.

[b55] Swindells J.F., Snyder C., Hardy R.C., Golden P. (1958).

[b56] Telis V.R.N., Telis-Romero J., Mazzotti H., Gabas A.L. (2007). Viscosity of aqueous carbohydrate solutions at different temperatures and concentrations. Int. J. Food Prop..

[b57] Thorat A.A., Forny L., Meunier V., Taylor L.S., Mauer L.J. (2018). Effects of mono-, di-, and tri-saccharides on the stability and crystallization of amorphous sucrose. J. Food Sci..

[b58] Van Der Sman R. (2013). Predictions of glass transition temperature for hydrogen bonding biomaterials. J. Phys. Chem. B.

[b59] Vesanen P.T., Zevenhoven K.C., Nieminen J.O., Dabek J., Parkkonen L.T., Ilmoniemi R.J. (2013). Temperature dependence of relaxation times and temperature mapping in ultra-low-field MRI. J. Magn. Reson..

[b60] Veser J., Kodde J., Groot S., Fix R., van der Sman R., Schutyser M. (2024). Universal or species-specific influence of moisture on the glass transition in various horticultural seeds?. Sci. Hort..

[b61] Woodbury T.J., Mauer L.J. (2022). Oligosaccharides elevate the gelatinization temperature of wheat starch more than sucrose, paving the way for their use in reduced sugar starch-based formulations. Food Funct..

[b62] Yoshioka S., Aso Y., Kojima S. (1999). The effect of excipients on the molecular mobility of lyophilized formulations, as measured by glass transition temperature and NMR relaxation-based critical mobility temperature. Pharm. Res..

[b63] Zielinski J.M., Benesi A.J., Duda J. (1992). Use of solvent carbon-13 relaxation to predict the temperature and concentration behavior of polymer/solvent diffusion coefficients. Ind. Eng. Chem. Res..

